# Ni-Supported Pd Nanoparticles with Ca Promoter: A New Catalyst for Low-Temperature Ammonia Cracking

**DOI:** 10.1371/journal.pone.0136805

**Published:** 2015-08-26

**Authors:** Jaroslaw Polanski, Piotr Bartczak, Weronika Ambrozkiewicz, Rafal Sitko, Tomasz Siudyga, Andrzej Mianowski, Jacek Szade, Katarzyna Balin, Józef Lelątko

**Affiliations:** 1 Institute of Chemistry, University of Silesia, Szkolna 9, 40–006 Katowice, Poland; 2 Department of Chemistry, Silesian University of Technology, 44–100 Gliwice, Poland; 3 A. Chełkowski Institute of Physics, University of Silesia, Silesian Center for Education and Interdisciplinary Research, 41–500 Chorzów, Poland; 4 Institute of Materials Science, University of Silesia, 75 Pułku Piechoty 1A, 41–500 Chorzów, Poland; RMIT University, AUSTRALIA

## Abstract

In this paper we report a new nanometallic, self-activating catalyst, namely, Ni-supported Pd nanoparticles (Pd_NPs_/Ni) for low temperature ammonia cracking, which was prepared using a novel approach involving the transfer of nanoparticles from the intermediate carrier, i.e. nano-spherical SiO_2_, to the target carrier technical grade Ni (t-Ni) or high purity Ni (p-Ni) grains. The method that was developed allows a uniform nanoparticle size distribution (4,4±0.8 nm) to be obtained. Unexpectedly, the t-Ni-supported Pd NPs, which seemed to have a surface Ca impurity, appeared to be more active than the Ca-free (p-Ni) system. A comparison of the novel Pd_NPs_/Ni catalyst with these reported in the literature clearly indicates the much better hydrogen productivity of the new system, which seems to be a highly efficient, flexible and durable catalyst for gas-phase heterogeneous ammonia cracking in which the TOF reaches a value of 2615 mmol_H2_/g_Pd_ min (10,570 mol_NH3_/mol_Pd(NP)_ h) at 600°C under a flow of 12 dm^3^/h (t-Ni).

## Introduction

Ammonia cracking is a method that is used in the treatment of flue gases from coal or biomass gasification or for hydrogen generation in chemical or related industries. It is an important issue in hydrogen economy. As a carbon-free compound, ammonia provides a potential source of ecological fuel for mobile and stationary power generation, especially for fuel cells in which CO impurities are unacceptable. Ammonia cracking is widely used in industry [1]; however, more efficient catalysts are needed for new technologies in this area, which is a difficult problem [2].

Ammonia decomposition is a complex process that proceeds through a stepwise dehydrogenation that yields H and N, which recombine into H_2_ and N_2_, respectively. Although the binding energy of the nitrogen must be sufficiently strong for dehydrogenation, it should not be so high that it blocks the recombination step [3–5].

Among the potential catalysts for this process, Ru has been observed to be the most active [6]; however, the effects of the Ru particle size and shape can be very difficult to elucidate and a simultaneous comparison of these factors is almost impossible [7]. Ni has been reported to exhibit an activity ca. 40% lower than Ru, whereas the activities of other metals have been reported to be more than three times lower [6]. The activities of various other metals have been reported in the following order Ru > Ir > Rh > Ni > Pt > Pd > Fe [6–8].

Structural manipulation of the catalysts for ammonia decomposition has been carefully reviewed elsewhere [7]. Because the cost of the catalysts for ammonia cracking is an important issue that limits their potential practical use, the substitution of Ru, in particular, by Ni appears to be an attractive option. For example, bimetallic Ni alloys, especially those that contain Mo, Co or Pt, have been tested as potential catalysts for this process. A catalyst that was prepared by the conventional (non-nano) combination of Pd/Ni exhibited a relatively low activity and had a conversion rate of 100% at 650°C vs. 600°C for Ru/Ni. These catalysts outperformed pure Ni, which required a temperature of 700°C for comparable activity [9]. Moreover, it has been established that, for various metals such as Fe, Co and Ni, nitrogen desorption limits the ammonia cracking rate, while the N-H bond scission step limits the reaction for Rh, Ir, Pd, Pt and Cu [6].

Nanotechnology is another option for the construction of Ni-based catalysts. In a study aimed at the potential applications of Ni nano-particles in ammonia cracking (Pd_NPs_), Zhang et al. established that the optimal size of Ni/Al_2_O_3_ and Ni/La–Al_2_O_3_ is 2.3 nm [10, 11]. The performance of this system, which was tested in a temperature range of 600–900°C, produced hydrogen production rates between 0 and 35 mmol H_2_ g_cat_^-1^ min^-1^ (up to 1,250 mol_NH3_/mol(Me_NP_) h) [6]. Monolayer Ni supported on Pt, Ru or tungsten monocarbide surfaces that was obtained via Ni vapor deposition has also been investigated [5, 12] and Pd/Ni nanoalloys with well-defined bimetallic compositions have been tested as CO oxidation catalysts [13]. When Pt-Ni bimetallics were investigated in catalytic ammonia cracking, an activity enhancement was observed, especially for Ni–Pt–Pt combination, which appeared to be the most active and compared well to the activity of the best Ru catalysts [5].

Because of the potential industrial importance of low-cost and efficient catalysts for ammonia decomposition, we began investigating the synergistic effects of Pd_NPs_ supported on Ni. Both metals have been determined to be highly active catalysts that influence the two complementary steps of this reaction; therefore, they can interact synergistically, thereby advantageously influencing the entire process. To the best of our knowledge, such a combination has never previously been tested. We assumed that directly contacting Pd_NPs_ to Ni grains would offer interesting capabilities in the production of industrial catalysts where the supporting Ni, e.g., in the form of wire meshes, could serve not only as a catalytic moiety but also as a flexible catalyst support.

Over the past few years, a number of techniques have been developed for the production of nanosized metallic particles and their distribution on different carriers. The methods that have recently been used, which are based on the “bottom-up” and the “top-down” approaches, still have some disadvantages, including the broad range of nanoparticle size distributions and a tendency to aggregate or to form clumps [14, 15]. To minimize these problems, we recently developed a novel, innovative method for the formation of a bimetallic catalyst that appears to be highly efficient in Sonogashira coupling [16] and glycerol oxidation [17]. Here, we show that this method can also be used for other bimetallic systems.

## Material and Methods

### Preparation of Pd_NPs_ on Ni

We prepared Pd_NPs_ supported on silica as an auxiliary source of nanoparticles. The silica was prepared using the Stöber method [18] with tetraethyl orthosilicate (TEOS), which was added to a mixture of ethanol and an aqueous ammonia solution. After silica separation, a solution of palladium precursor (PdCl_2_) was added. The mixture was sonicated, then concentrated, dried and reduced under hydrogen at 500°C. In a typical procedure, 800 mL of anhydrous ethanol and 135 mL of a 25 wt.% solution of ammonia were mixed with 78 mL of deionized water. After 10 min of stirring, 60 mL of tetraethyl orthosilicate was added to the reaction mixture, which was next stirred for 3 h at room temperature. The colloidal silica suspension that was obtained was centrifuged, washed to neutral pH (deionized water) and suspended in deionized water (20 mL) in an ultrasound bath and stirred for 90 min. A solution containing palladium precursor (273 mg palladium(II) chloride for 1% Pd/SiO_2_) in deionized water (30 mL) was added dropwise into the colloidal silica suspension and mixed in an ultrasound bath for 30 min. Next, it was dried to a constant weight at aprox. 90°C, ground and sieved. The reduction was conducted in an oven under hydrogen at 500°C for 4 h. Alternatively, we prepared Ru NPs supported on silica using 424 mg RuCl_3_ hydrate (36.6% Ru, Acros Organics) as Ru precursor using this procedure.

Bimetallic Pd_NPs_ /Ni catalyst was prepared using a novel approach involving the transfer of nanoparticles from the intermediate carrier, i.e. SiO_2_, to the target carrier. The general method includes several steps. The target carrier, i.e. Ni, (0.99 g) and Pd_NPs_ of a low polydispersity deposited on the intermediate carrier, i.e. 1.0% Pd_NPs_/SiO_2_, (1.00 g) were suspended in deionized water (100 mL) under mechanical stirring and sonication. After 10 minutes of vigorous stirring, sodium hydroxide (40 mL 40% w/w) was added to the suspension and stirring was continued for 4 h at 80°C, whereupon the suspension was allowed to stand for about 18 h until the suspended solids sedimented. The suspension was centrifuged and the supernatant was decanted, the precipitate was washed in deionized water and centrifuged again to achieve a neutral pH of the supernatant. The precipitate was washed with deionized water once more, centrifuged and the supernatant was removed. The catalyst that was obtained was dried in an electric dryer to a constant weight at 110°C. To characterize the pore structure, a 3Flex, produced by Micromeritics, USA, was used to determine the N_2_ adsorption isotherm at 77 K in the range of 0.05 to 0.3 relative pressure in order to calculate the BET surface area. Prior to the measurement, the sample was degassed in a vacuum at 350°C for 5 h. In Table 1 we specified the surface area of the catalysts and/or other systems tested.

**Table 1:** Specific surface area of the catalysts, precursors and reference materials investigated.

System	Specific Surface Area [m^2^/g]
Pd/t-Ni	120.5
Pd/p-Ni	209.1
Pd/SiO_2_	187.4
Ru/SiO_2_	203.8
t-Ni	115.3
p-Ni	147.0
PdO	68.2
t-Ni/SiO_2_ ^a^	132.1
t-Ni processed^b^	89.7

a/ a blind t-Ni sample processed similarly to bimetallic Pd/t-Ni with SiO_2_ but without Pd

b/ a blind t-Ni sample processed similarly to bimetallic Pd/t-Ni but without SiO_2_ and Pd

The chemical analysis of nano-materials was performed using an energy-dispersive X-ray fluorescence (EDXRF) spectrometer—Epsilon 3 (Panalytical, Almelo, The Netherlands) with a Rh target X-ray tube operated at the max. voltage of 30 keV and max. power of 9W. The spectrometer is equipped with a thermoelectrically cooled silicon drift detector (SDD) with an 8μm Be window and a resolution of 135 eV at 5.9 keV. The quantitative analysis was performed using Omnian software based on the fundamental parameter method and Pd Lα line (2.84 keV). The chemical analysis was also performed using a laboratory-constructed EDXRF spectrometer equipped with a Rh target X-ray tube operated at the max. voltage of 50 keV and max. power of 75W (XTF 5011/75, Oxford Instruments, USA). The X-ray spectra were collected using a thermoelectrically cooled Si-PIN detector with a 12.5μm Be window and 145eV resolution at 5.9 keV (XR-100CR Amptek, Bedford, MA, USA). The deconvolution of X-ray spectra and quantitative analysis were performed using XRF-FP Amptek software and the Pd Kα line (21.18 keV).

### Ammonia cracking

Ammonia cracking was performed under atmospheric pressure in a quartz flow microreactor with a fixed catalyst bed that had a diameter of 7.5 mm, 0.097 cm^3^ by volume (2.2 mm height). The feeding gas, NH_3_, was continuously injected at a flow rate of 2 dm^3^/h (350 kg/h kg_met_ in relation to the Pd_NPs_). Alternative flows ranged from 2–12 dm^3^/h. Ammonia was provided by Zakłady Chemiczne POLICE, Poland; the guaranteed purity was 99.8%. The NH_3_ conversion was determined by analyzing the composition of the tail gas effusing from the microreactor using a thermal conductivity detector-equipped SRI gas chromatograph (1/8 inch diameter, 3-m-long column; micropacked with active carbon 80–100 mesh; column temperature of 80°C, with Ar as the effluent gas, 10 dm^3^h^-1^). We illustrated the reaction by ammonia consumption; however, the nitrogen and hydrogen formation were also monitored in order to balance the reaction. At the same time, we also determined the potential remains of ammonia in the stream of the products. Ammonia is tolerated for some fuel cells but occasionally contamination will poison the system. In such cases in order to avoid the equilibrium limitation, removal of the hydrogen through a membrane is an option that would provide a pure hydrogen feed that is free of ammonia and nitrogen, and this would help to increase the conversion by shifting the equilibrium further towards complete conversion [19]. The potential remains of ammonia are given by the detection threshold of GC, which is 1 ppm. The standard deviation of the ammonia conversion degree amonuted to 0.32% (5 measurements).

## Results and Discussion

### The catalysts preparation and structure

**Fig 1:**
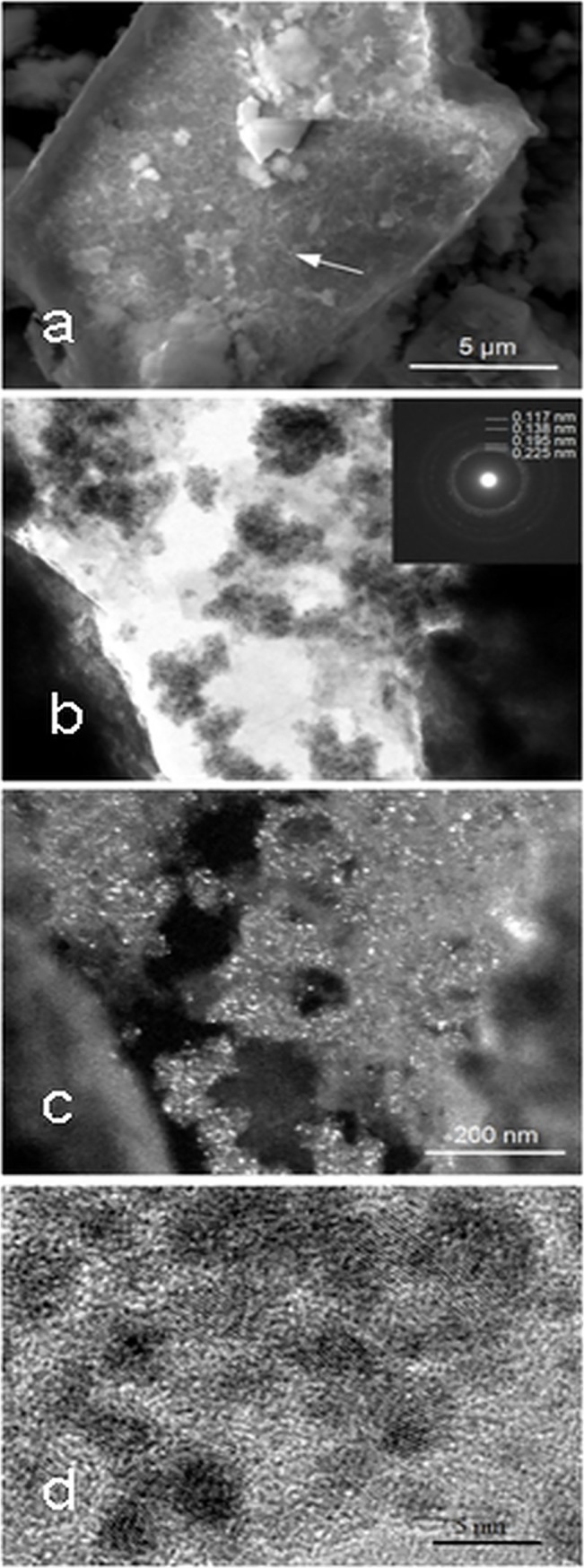
Representative SEM and TEM images of the Pd/Ni catalyst (a-c: t-Ni; d: p-Ni system). a—SEM image of the Pd NPs aggregates (e.g., indicated by arrows) on the Ni surface; b—TEM bright field image containing the electron diffraction pattern of Pd particles in the corner, c—TEM dark field images of the Pd nanocrystalline aggregates on the Ni surface; d—HRTEM image of the Pd NPs.

Bimetallic Pd/Ni contacts were obtained via sonication of Pd_NPs_/SiO_2_ and nickel powder and the subsequent digestion of silica with 40% aqueous NaOH. This was washed with deionized water to neutral pH and dried to provide the final catalyst. The morphology and composition of the resulting bimetallic Pd/Ni system were studied using scanning electron microscopy (SEM), transmission electron microscopy (TEM), X-ray photoelectron spectroscopy (XPS) and energy-dispersive X-ray fluorescence (EDXRF) spectroscopy. We used technical (t-Ni) and high purity nickel (p-Ni) carriers to prepare the catalysts. In Fig 1 we show the structure of the final catalysts with Pd_NPs_ mounted on the Ni grains. The average sizes of the supported Pd nanoparticles were determined by analyzing data from different TEM (Fig 1D) images, and showed an average diameter of 4.4 ± 0.8 nm. The detailed size distribution analysis is presented as supplementary data.

XPS and EDXRF analyses were used to investigate the nature of the Pd and Ni surfaces and contacts. XPS indicated that Pd and Ni oxides (in particular, PdO and Ni_2_O_3_) dominated the surface structures; however, unoxidized Pd and Ni were also observed as is detailed in entry 1 of Table 2, which indicates the ratio of the two components. In the case of Pd 3d_5/2_, the line with the maximum at 336.6 eV (t-Ni) or 336.8 eV (p-Ni) corresponds to PdO and the one that is situated at 335.4 eV (t-Ni) or 335.5 eV (p-Ni) can be attributed to metallic Pd. In the case of the Ni 2p_3/2_, the ratio was calculated from the intensities of the lines that originated from Ni_2_O_3_ (t-Ni: 855.9 eV; p-Ni: 856.1 eV) and metallic Ni (t-Ni: 852.45 eV; p-Ni: 852.7 eV), although XPS analyses cannot conclusively rule out the formation of PdNi because small chemical shifts in these alloys [20] make determinations of the contributions from the pure metals and potential alloys doubtful. However, the low-temperature catalyst preparation means that metal alloying is unlikely.

**Table 2:** XPS peak positions (binding energy in eV) and relative intensity ratios of the metallic and oxidized components of the nano-Pd/Ni (t-Ni) catalyst before (BR) and after (AR) reaction.

Sample		XPS				EDXRF^e^
		Pd(Ni)/PdO^a^	(Pd)Ni/Ni_2_O_3_ ^b^	Pd/Ni^c^	Pd/SiO_2_ ^d^	Pd/Ni^d^
1	BR^f^	0.64	0.05	0.31	0.56	0.026 (0.013)
2	AR^g^	2.59	0.28	0.04	0.35	0.024 0.011)

a/ Pd 3d_3/2_: ratio of metallic (335.4) Pd(Ni) to oxidized PdO (336.6). Pd(Ni) means pure metallic Pd or Pd-Ni alloy.

b/ Ni 2p_3/2_: ratio of metallic (Pd)Ni (852.45) to oxidized Ni_2_O_3_ (855.9). (Pd)Ni means pure metallic Ni or Pd-Ni alloy.

c/ Based on atomic weights, Pd = 106.42, Ni = 58.69; if based on atomic contributions, the Pd/Ni ratio is 0.17. Total intensity of the Pd 3d and Ni 2p XPS lines was taken for the calculation.

d/ Based on atomic weights.

e/ Information depth of 4 μm (unbracketed value) or 60–85 μm (bracketed value), respectively. The information depth d_99%_ for element *i* that would yield 99% of the element intensity is given by the formula d_99%_ = 4.6 / χ(E_0_,E_i_) × ρ, where ρ is the density of the sample and χ(E_0_,E_i_) = μ(E_0_)csc(ϕ_1_) + μ(E_i_)csc(ϕ_2_) is the total mass-attenuation coefficient of the sample. Variables μ(E_0_) and μ(E_i_) represent the mass attenuation coefficients of the sample at the primary *E*_0_ and fluorescent radiation *E*_i_ (Pd-Lα or Pd-Kα line at 2.84 or 19.28 keV, respectively); ϕ_1_ and ϕ_2_ are the incidence and take-off angles, respectively.

f/ Catalyst sample before the reaction.

g/ Catalyst sample after 200 h of processing at temperatures up to 650°C.

XPS, when used to determine of the Pd/Ni ratio (Table 2, entry 1, column 5), gave a value that was approximately 30 times greater than the expected Pd/Ni value of 0.01, which is easily understood when we realize that the photoelectron escape depth is approximately 4–5 nm. Thus, Pd deposited on the surface is overrepresented in relation to the bulk catalyst composition. XPS also reveals the presence of the residual debris of silica at the surface (Table 2, entry 1, column 6), which may preserve some of the original Pd_NP_/silica forms and help avoid NP agglomeration.

EDXRF analysis was performed to further prove the chemical composition of the catalyst. The Pd concentration, which was determined using the Pd-Lα line and a Rh-target X-ray tube operated at a maximum voltage of 30 keV, indicated that the catalyst had a Pd concentration of 2.43% or a Pd/Ni ratio 0.026 (Table 2, entry 1, column 7). However, the low energy from the Lα line resulted in a low information depth d_99%_ of ca. 4 μm (Table 2: footnote e), which resulted in an effect that was similar to the one that was encountered during the XPS analysis, thus indicating a surface Pd concentration that was higher than expected for the bulk composition. However, in comparison with the XPS analysis, the EDXRF (Pd Lα) analyses indicated an approximately ten-fold lower result. The substantially greater energy that was used in EDXRF (Pd-Kα line) resulted in an increased penetration depth of the X-rays to d_99%_ = 60–85 μm, which was now comparable to the size of the t-Ni grains (diameter less than 50 μm). In this case, the determined Pd concentration (ca. 1.2%) or Pd/Ni ratio of 0.013 (Table 2, entry 1a) was close to the value that was expected for the bulk composition.

### Ammonia cracking

**Fig 2:**
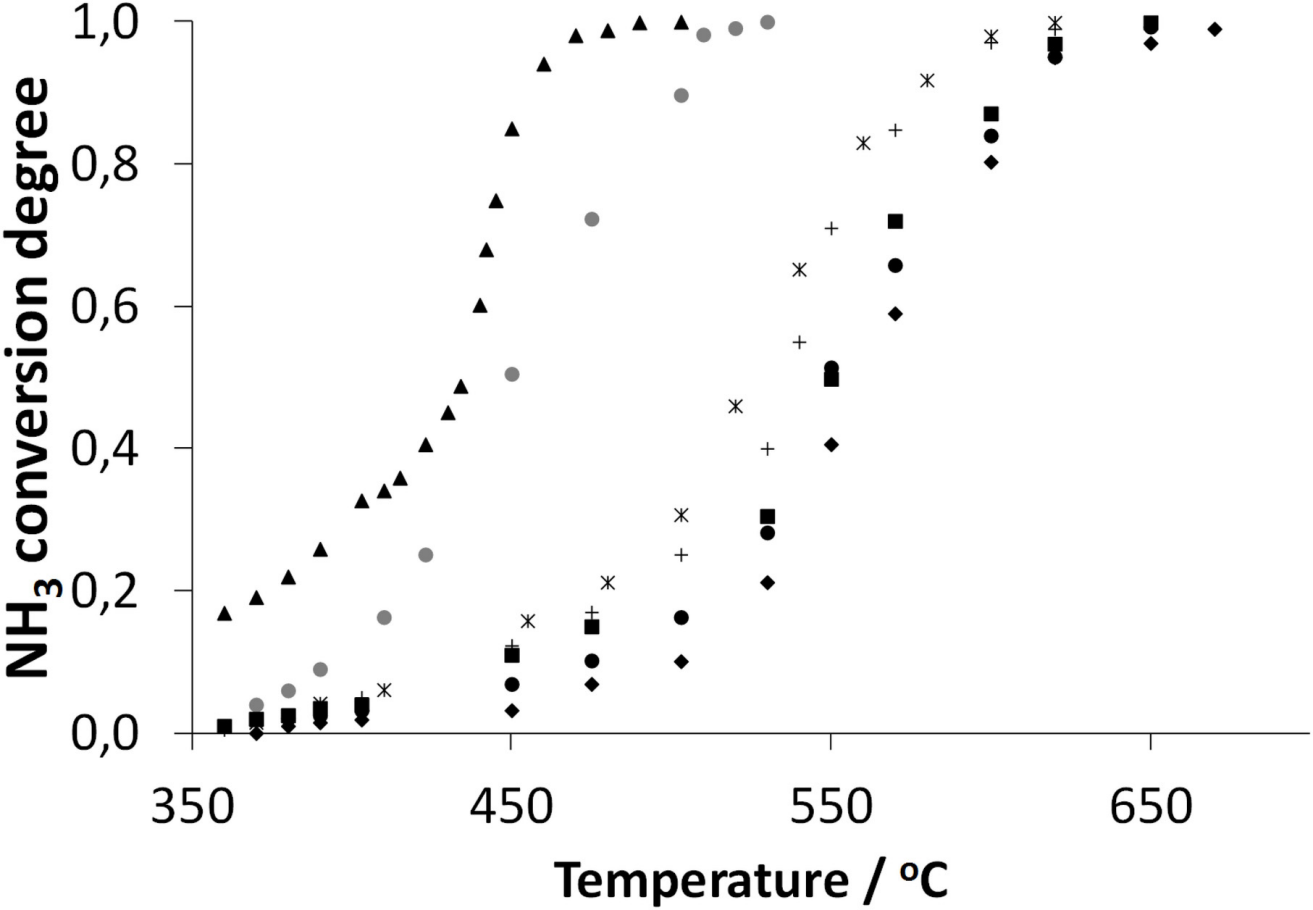
Ammonia conversion on the Pd/Ni catalyst for the p-Ni (shadowed circles) and t-Ni (black triangles) systems, compared with that on the analogously processed control t-Ni carrier without Pd and SiO_2_ (black squares), analogously processed control t-Ni without Pd but with SiO_2_ (diamonds), unprocessed t-Ni (black circles), PdO (crosses) and Pd/SiO_2_ (asterisks) at a flow rate of 2 dm^3^/h.

Fig 2 shows a representative set of ammonia cracking data for the novel catalysts supported on t-Ni and p-Ni carriers. The data were fully reproducible when repeated for independently synthesized catalyst samples. In our experiments, the temperatures that were needed for the complete conversion of ammonia were in the range 450–600°C, depending on the ammonia flow rates. We compared the activity of the Pd/Ni system to the activities of a series of reference materials, in particular, a control sample of supporting t-Ni grains and t-Ni grains that were prepared in a manner similar to the Pd/Ni system but without the addition of Pd (Fig 2). All of the systems that were tested were significantly less reactive than the Pd_NPs_ supported on Ni. In particular, the activity of the bimetallic Pd/Ni system clearly exceeds that of pure Ni, which required ca. 650°C to achieve 100% conversion. The latter compares well with previously reported results for a similar Ni system that needed 700°C for full conversion [9].

Ru catalysts were supported on various carriers and investigated as promising ammonia-cracking systems [21–23]. Therefore, in Table 3, we compared the hydrogen productivity as given by the turnover frequency (TOF) for the Ru catalysts data available in the literature [21–23], i.e., Ru/SiO_2_ (Table 3: columns 5–6) or Al_2_O_3_ (Table 3: column 7), and the TOF value for new bimetallic Pd/Ni catalyst (Table 3: column 2). Additionally, in Table 3 we are including the TOF data for the Pd/SiO_2_ system that was used as the precusor for our bimetallic Pd/Ni system and for the Ru/SiO_2_ system which we synthesized just for the comparison. This analysis demonstrates that the hydrogen productivity for our novel Pd_NPs_/Ni system compares advantageously with the Ru catalysts that are believed to form the most active system.

**Table 3:** Comparison of the TOFs for the investigated Pd_NPs_/Ni (Pd_NPs_/SiO_2_) and the most reactive Ru systems [21–23].

T [°C]	TOF^a^ [mmol_H2_/g_met_ min]					
	Pd/Ni (1% Pd)^b^ ^,^ ^c^	Pd/SiO_2_ (1% Pd)^b^	Ru/SiO_2_ (1% Ru)^b^	Ru/SiO_2_ (10% Ru)^[^ ^21^ ^]^	Ru/SiO_2_ [22]^,^ ^d^	Ru/Al_2_O_3_ (5% Ru)^[^ ^23^ ^]^
400	143.3//55.4	18.8	0.0	4.5	16.5	12.5
450	372.4//221.2	52.9	2.7	11.4	32.9	39.6
500	437.9//392.9	108.3	8.7	20.0	43.2	117.0
550	not tested	261.5	186.3	not tested	48.1	240.0
600	not tested	345.8	326.6	30.3	48.1	not tested
650	not tested	not tested	not tested	30.9	48.1	not tested

a/ TOF units as reported for the literature data for a flow of 3 dm^3^/h [21–23]; if calculated for NH_3_ (data in text), TOF = Vα / n, where V is the molar flow rate of NH_3_, α is the conversion degree and n is the moles of Me NPs (Me_NP_).

b/ investigated in this study

c/ Pd/t-Ni**//**Pd/p-Ni at a flow rate of 2 dm^3^/h; TOF/T[°C] amounted to 120/400, 302/450, 1235/500 for a flow rate of 6 dm^3^/h or 79/400, 237/450, 1370/500, 2384/550, 2615/600 for a flow rate of 12 dm^3^/h, respectively.

d/ The Si/Ru ratio was 0.2; BET surface area—42 m^2^/g.

The high activity of the new catalyst can additionally be highlighted if we realize that under the current experiment in flow (short contact time reactor), the activity of all reference systems was flattened, i.e. the observed activity was very similar, as can be observed in Fig 2. This can be explained two ways.

First, it was observed previosuly for the Ru/SiO_2_ system that the “spacing of the SiO_2_^”^ and the “enhancement in the exposure of the Ru” have a “positive effect on catalytic performance” [22]. Thus, the hindered availability of the catalyst for the reacting ammonia, e.g., by the location of the metal NPs in the carrier pores, will cause a problem of a mass transfer limitation. This should result in differences in the catalyst activity depending on the structure of the individual catalysts that have a different mass transfer resistance. In fact, if we compare the literature data for Ru catalysts on SiO_2_ (Table 3: columns 5–6), we can see a relatively large deviation in the TOF values, particularly at lower temperatures, e.g., the TOF value ranges from 4.5 to 16.5 mmol_H2_/g_met_ min at 400°C (Table 3: column 5 vs. 6). This effect is less important at higher temperatures, e.g., at 650°C the difference in the TOF values amounts to 30.9 vs. 48.1 mmol_H2_/g_met_ min (Table 3: column 5 vs. 6). This is what should be expected, the lower the temperature of the reaction is the more important mass limitation is due to the lower penetration ability of the reacting gases.

A similar effect can explain the relatively low activity of the Ru_NPs_/SiO_2_ system that we prepared and tested in our reactor in order to obtain a more reliable comparison (Table 3: column 4) when compared to literature data (Table 3: columns 5–6). Actually, we previoulsy observed an activity depression for the SiO_2_-supported nano metal catalysts that was obtained using the current method, where metal NPs were hindered in the silica pores [17]. Similarly to the literature Ru systems, the mass transfer limitation revealed here appeared to be less important with an increase in the temperature of the ammonia cracking where the reactants can penetrate the silica pores more easily, e.g., the TOF value for the 1% Ru/SiO_2_ catalyst (Table 3: column 4) at 600°C is higher than the most active 10% Ru/SiO_2_ system (Table 3: column 5) resembling this for the Ru/Al_2_O_3_ (Table 3: column 7). In our investigations 1% Pd/SiO_2_ system (Table 3: column 3) appeared more reactive than the 1% Ru/SiO_2_ system (Table 3 column 4), especially at lower temperatures.

The BET surface area (Table 1) of the catalysts that were tested changed as follows—203.8 m^2^/g (Ru/SiO_2_); 187.4 m^2^/g (Pd/SiO_2_); 120.5 m^2^/g (Pd/t-Ni). This indicated a difference between the SiO_2_-supported systems (surface area amounted to ca. 200 m^2^/g) and Pd/t-Ni (120.5 m^2^/g). The relatively large, low porosity t-Ni grains, which are used as a catalyst carrier, determine the availability of the Pd_NPs_ that is supported by t-Ni. In turn, in the Pd/SiO_2_ system NPs sit inside the SiO_2_ pores and consequently during ammonia cracking, they are much less available and their activity is lower (Table 3: column 3).

Secondly, however, the availability of Pd on the Ni carrier and mass transfer effects cannot fully explain the high activity of the Pd/Ni system because low activity was observed when the Ni grains were tested without Pd NPs or Pd(O) without Ni (Fig 2). At the same time, the Pd/p-Ni of the higher surface area (209.1 m^2^/g) was less reactive than the Pd/t-Ni system, but still much more reactive than the pure Pd or Ni systems (Fig 2). This indicates that synergistic Ni/Pd interactions are required under the current experimental conditions for high catalyst activity.

Short contact also determines that the systems are still far from thermodynamic equilibrium (compare supplementary materials for calculations). On the other hand, the temperature was carefully stabilized before the measurements were performed at a certain temperature. Thus, the reaction took place under the conditions when heat transfer was not a limiting factor.

Unexpectedly, the t-Ni powder that we originally used for the catalyst preparation (coded by filled triangle in Fig 2) included up to ca. 3% of Ca (indicated by EDXRF and XPS) on the surface and the additonal ingredient may be an interesting source of synergy in catalytic systems [24]. Interestingly, alkali metals that had been absorbed into a silica gel were reported as reducing agents and hydrogen source [25]. Accordingly, in order to conclusively prove the role of Ca, we prepared the catalyst on p-Ni that was proven not to have any Ca contamination by both XPS and EDXRF. These results are shown in Fig 2. Thus, the highest activity at the lowest temperatures was observed for the catalyst that was prepared for t-Ni with the highest Ca content. For high purity Ca free p-Ni based catalyst we observed lower activity which was, however, higher than the activity for unsupported Ni or Pd systems. The activation energy that was calculated for the t-Ni and p-Ni catalysts amounted to 59 kJ/mole and 64.1 kJ/mole, respectively (compare supporting information for calculations). A comparison of hydrogen productivity for the Ca and Ca-free systems (Table 3, column 2), especially at low temperatures, indicated a large difference in the advantage of the Ca system, i.e. at 400°C TOF Ca: ca. 143 mmol_H2_/g_Pd_ min (579 mol_NH3_/mol_Pd_ h) vs. TOF Ca free: 55 ca. mmol_H2_/g_Pd_ min (224 mol_NH3_/mol_Pd_ h). This effect is much less pronounced at higher temperatures (Table 3, column 2).

A question may be asked about the influence of the surface area of the t-Ni vs. p-Ni catalysts (120.5 m^2^/g (Pd/t-Ni) vs. 209.1 m^2^/g (Pd/p-Ni)). Although the surface area value of the Pd/p-Ni system is close to this of the the Pd/SiO_2_ one, the Pd is now mounted on the Ni surface in the form of solid NPs and not those that were reduced from the liquid phase, which were capable of penetrating the pores of the supporting SiO_2_ in the Pd/SiO_2_ system. Thus, we can clearly see visible Pd NPs on the surface of p-Ni in the TEM microphotographs (Fig 1D) and the difference in the surface area of the t-Ni vs. p-Ni system results from the differences of the Ni surfaces, as can be compared in Fig 1A vs S2A Fig.

**Fig 3:**
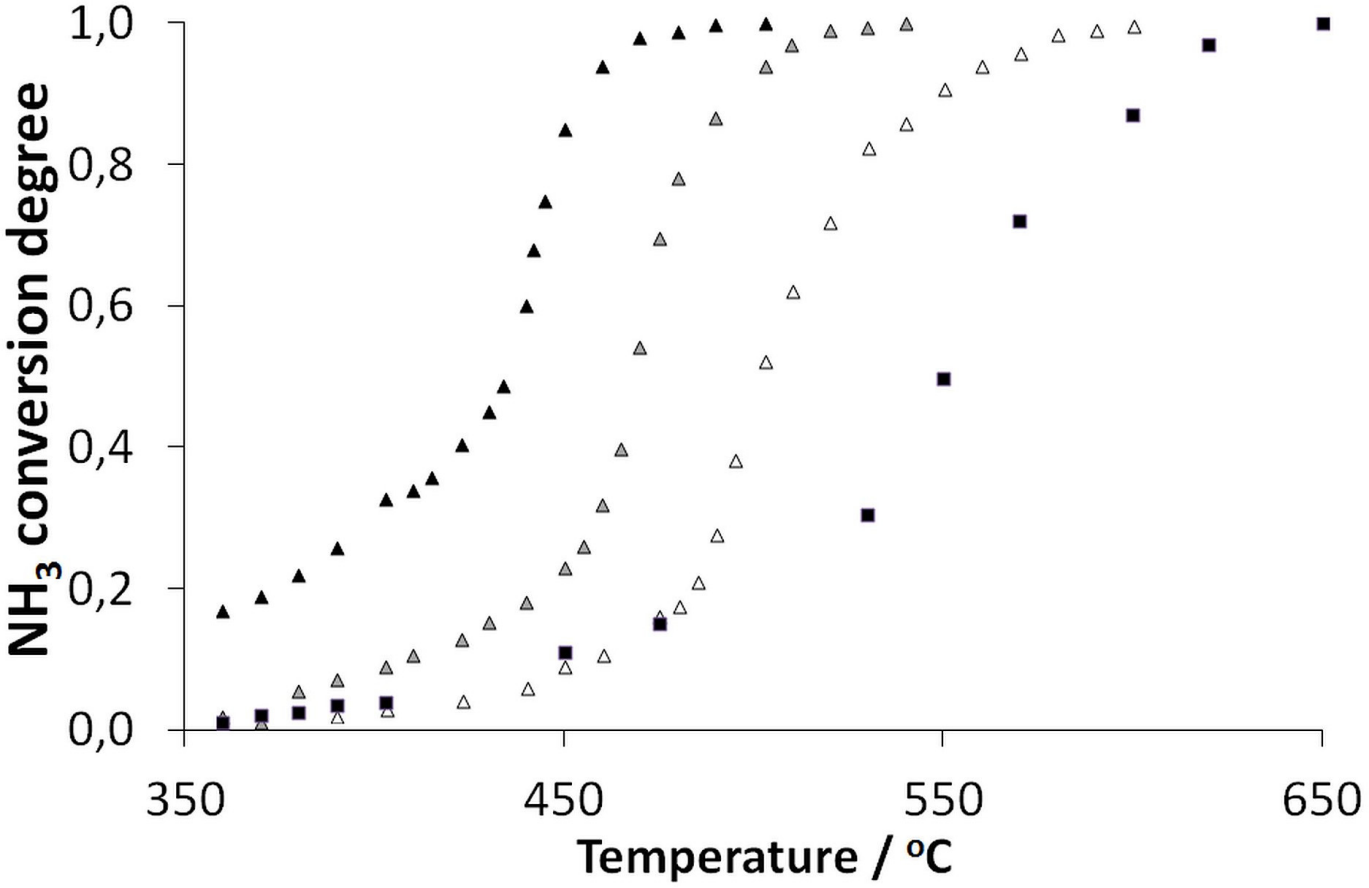
Ammonia conversion on the Pd/Ni (t-Ni catalyst) for different ammonia flow rates of 2 dm^3^/h (black triangles), 6 dm^3^/h (shadowed triangles) or 12 dm^3^/h (white triangles) compared with the control Ni carrier that was preprocessed analogously (but without Pd NPs) at a flow rate of 2 dm^3^/h (black squares).

Fig 3 shows a comparison of the ammonia cracking data for the novel t-Ni catalyst that was registered for different ammonia mass flows ranging from 2 to 12 dm^3^/h. The TOF can reach a value of 2615 mmol_H2_/g_**Pd**_ min (10,571 mol_NH3_/mol_Pd_ h) at 600°C under a flow of 12 dm^3^/h. A comparison of the behavior of the raw catalyst after pre-activation via a reduction of hydrogen (data not shown) is also interesting. The pre-reduction induced no difference in the catalytic activity, which indicates that during ammonia cracking in the presence of the raw nano-Pd/Ni catalyst even at low temperatures, hydrogen is formed in a quantity that is sufficient for the immediate reduction of the small amount of the catalyst used. This self-activation via metal reduction is evident from the comparison of the catalyst structure before and after the reaction, which indicates much higher free metal to metal oxide ratio (Pd or Ni). After the reaction with t-Ni, the contribution from the metallic state (i.e. the lower binding energy line (335.4 eV) attributed to metallic Pd in the XPS spectrum) dominated vs. the PdO line (336.6 eV). These values are compared in columns 3 and 4 of Table 2. Interestingly, the same effects were detected for the Ni 2p multiplet for which the peak in the spectrum that was dominated by the oxide decreased in intensity relative to the one that was associated with the metallic state (582.45 eV). The same effect was observed for the p-Ni catalyst. Notably, a similar self activation by *in situ* hydrogen formation was previously observed for Ni-based catalysts [26].

In an additional series of experiments, we tested the stability of the catalyst during long-term ammonia processing. After 200 h under duty at 400°C, the performance of the Pd/p-Ni catalyst decreased ca. 0.27%. In turn, the t-Ni based catalyst operated at a much larger temperature amplitude up to 700°C, which indicated a conversion decrase from 100% to 98.76%, i.e. ca. 1% (compare supplementary materials, S13 Fig).

SEM and TEM images of the catalyst after this experiment are shown in the supplementary materials. Interestingly, when we used XPS analysis to compare the surfaces of the catalyst before and after the reaction, we observed a large discrepancy in the Pd-to-Ni ratio (estimated for the total amount of Pd and Ni) from the XPS and EDXRF data before and after the reaction. For example, for t-Ni system, according to XPS data, the Pd/Ni ratio changed significantly during the reaction (Table 2: column 5) and was 0.04 after the reaction (vs. 0.31 for a raw catalyst). In comparison, similar EDXRF data indicated practically no changes in the Pd/Ni ratio (Table 2: column 5). By changing the EDXRF penetration energy (footnote e to Table 2), we determined that the Pd/Ni ratio depends on the information depth. Thus, the changes in the Pd/Ni ratio are confined to the surface region and can only be observed using XPS, which cannot penetrate the full catalyst depth. On the other hand since the Pd/Ni ratio takes the account of the total amount of Pd and Ni and not only their metallic forms the changes cannot be explained by a simple enrichment of the surface in the metallic Ni form resulting from the reduction of the oxidized form of the dominating surface ingredient in the presence of the tiny amount of Pd (ca. 1%). Therefore, the fact that during the reaction Pd tend to be isolated from the very surface (XPS) but was preserved in the catalyst (EDXRF) need some other explanation. A comparison of the Pd/SiO_2_ ratio using XPS (Table 2: column 6) also shows changes in this indicator, which decreased at the very surface during the reaction, thereby indicating the enrichment of the surface layer in silica. Actually, silica sintering in the presence of other elements, e.g., amorphous Pd-Ni-Si alloys, has been observed at relatively low temperatures [27, 28]. Interestingly, these changes at the catalyst surface did not result in any important changes in the catalyst activity, which corresponds well to the generally high catalytic activity of silica encapsulated NPs [17, 29].

## Conclusions

In summary, we have developed a novel strategy for supporting nanoparticles on metal carriers. In particular, we prepared Pd_NPs_ supported on Ni, which appeared to be a highly efficient, self-activated, flexible and durable catalyst for gas-phase heterogeneous ammonia cracking, which is an important and still unsolved issue in the hydrogen economy.

Here, we showed that a combination of Pd_NPs_ on Ni, which was obtained using a novel method, resulted in synergistic effects for ammonia cracking. Moreover, we observed a promoting role of the Ca contamination that is found in t-Ni. The comparison of the TOF of the new catalyst with those that have been reported in the literature clearly indicates much better hydrogen productivity in the new system. Direct contact between Pd_NP_ to Ni grains also provides interesting possibilities for the potential production of low-cost and efficient industrial catalysts that use supporting Ni, e.g., in the form of wire meshes, which could serve not only as a component of the catalyst but also as a flexible catalyst support.

## Supporting Information

S1 Fig EDXRF spectrum. EDXRF spectrum of the Pd/Ni that was collected using an Rh target X-ray tube operated at 45kV and 300μA.
(TIF)

S2 Fig Representative SEM and TEM images of the Pd/Ni catalyst. Representative SEM and TEM images of the Pd/Ni catalyst for a p-Ni system: SEM (a), TEM bright (b) and dark (c).
(TIF)

S3 Fig XPS analysis of the t-Ni-based catalyst. Representative XPS analysis of the t-Ni-based catalyst before (blue) and after 200 hours of ammonia processing (red).
(TIF)

S4 Fig SEM image after ammonia cracking. SEM image of the catalyst after 200 hours of ammonia processing (Pd/t-Ni)
(TIF)

S5 Fig TEM image of the t-Ni-based catalyst after 200 hours ammonia processing. TEM image of the t-Ni-based catalyst after 200 hours of ammonia processing. The observed surface morphology indicates that Pd NPs are hiding between Ni agglomerates (a) or forming separate conglomerates with Ni or Ca (b, c).
(TIF)

S6 Fig XPS profile for the t-Ni-based system: Pd 3d. The reconvolution of the XPS profile (peak fitting) for the t-Ni-based system before the reaction.
(TIF)

S7 Fig XPS profile (peak fitting) for the t-Ni-based system: Pd 3d. The reconvolution of the XPS profile (peak fitting) for the t-Ni-based system after the 200 hour reaction.
(TIF)

S8 Fig XPS profile (peak fitting) for the t-Ni-based system: Ni 2p_3/2_. The reconvolution of the XPS profile (peak fitting) for the t-Ni-based system before the reaction.
(TIF)

S9 Fig XPS profile (peak fitting) for the t-Ni-based system: Ni 2p_3/2_. The reconvolution of the XPS profile (peak fitting) for the t-Ni-based system after the 200 hour reaction.
(TIF)

S10 Fig Pd NPs size distribution. Histogram of the size distribution of the Pd nanoparticles in the Pd/Ni catalyst
(TIF)

S11 Fig Thermodynamic equilibrium plot. Ammonia conversion on the Pd/t-Ni catalyst compared to the thermodynamic equilibrium (solid line) at a flow rate of 2 dm^3^/h.
(TIF)

S12 Fig Activation energy. Activation energy calculated for the catalyst and reference systems that were tested.
(TIF)

S13 Fig Conversion in long duration experiment. Conversion degree for a long duration experiment (Pd/t-Ni system).
(TIF)

S1 Text Reagents. Reagent list.
(PDF)

S2 Text EDX Spectra. EDX Spectra.
(PDF)

S3 Text Calculations of thermodynamic equilibrium. Calculations of thermodynamic equilibrium.
(PDF)
