# Supplementary figures and images for: Ni-Supported Pd Nanoparticles with Ca Promoter: A New Catalyst for Low-Temperature Ammonia Cracking
Source: PLoS One. 2015 Aug 26;10(8):e0136805. doi: 10.1371/journal.pone.0136805 (PMC4550460; doi:10.1371/journal.pone.0136805)

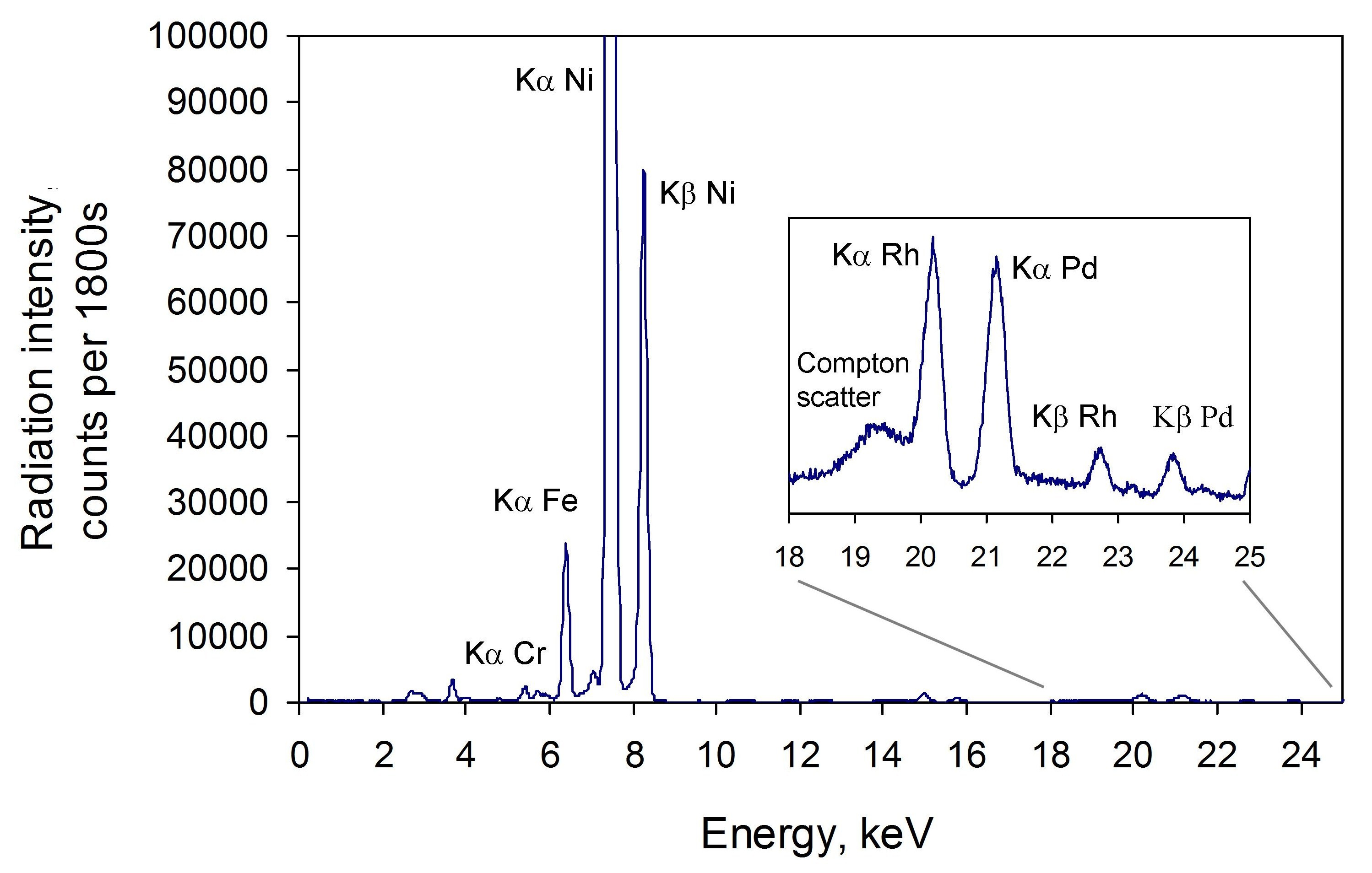

Supplement: S1 Fig — EDXRF spectrum of the Pd/Ni that was collected using an Rh target X-ray tube operated at 45kV and 300μA. (TIF) [file pone.0136805.s001.tif]

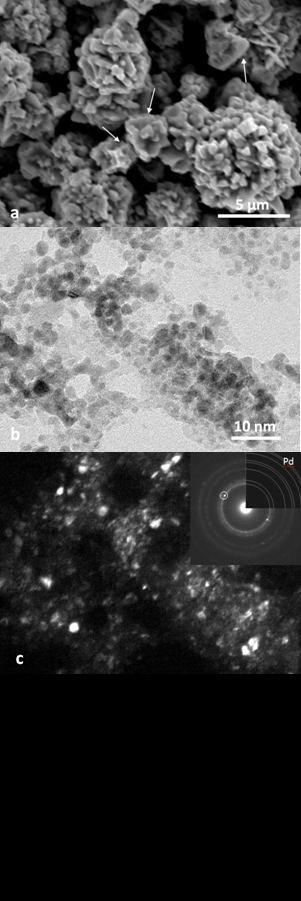

Supplement: S2 Fig — Representative SEM and TEM images of the Pd/Ni catalyst for a p-Ni system: SEM (a), TEM bright (b) and dark (c). (TIF) [file pone.0136805.s002.tif]

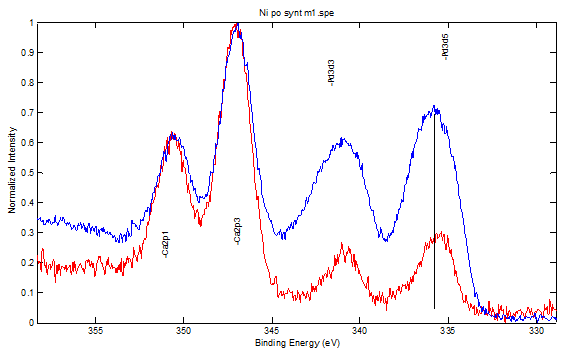

Supplement: S3 Fig — Representative XPS analysis of the t-Ni-based catalyst before (blue) and after 200 hours of ammonia processing (red). (TIF) [file pone.0136805.s003.tif]

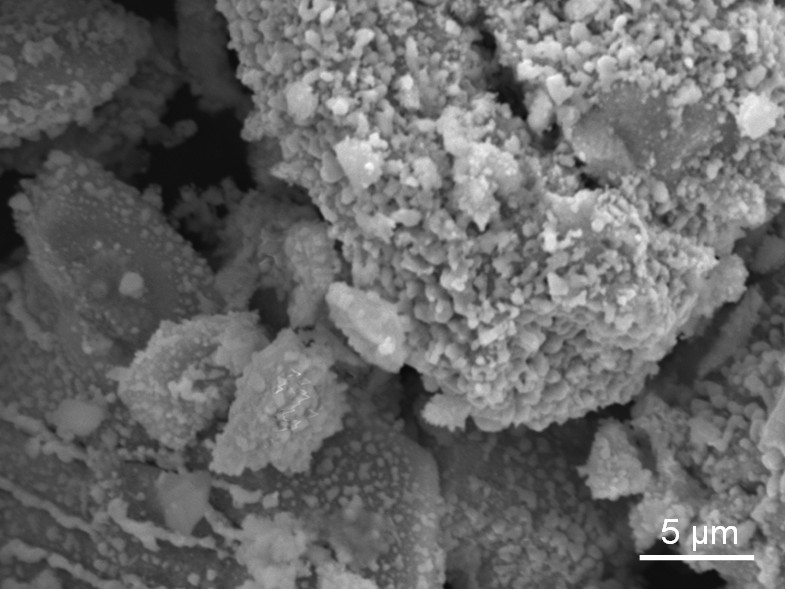

Supplement: S4 Fig — SEM image of the catalyst after 200 hours of ammonia processing (Pd/t-Ni) (TIF) [file pone.0136805.s004.tif]

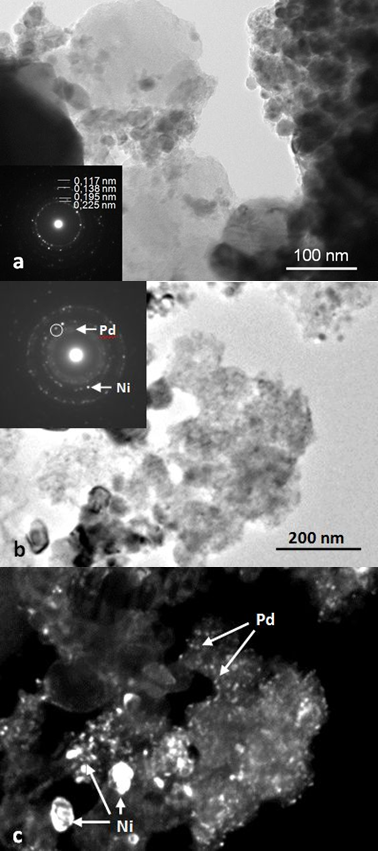

Supplement: S5 Fig — TEM image of the t-Ni-based catalyst after 200 hours of ammonia processing. The observed surface morphology indicates that Pd NPs are hiding between Ni agglomerates (a) or forming separate conglomerates with Ni or Ca (b, c). (TIF) [file pone.0136805.s005.tif]

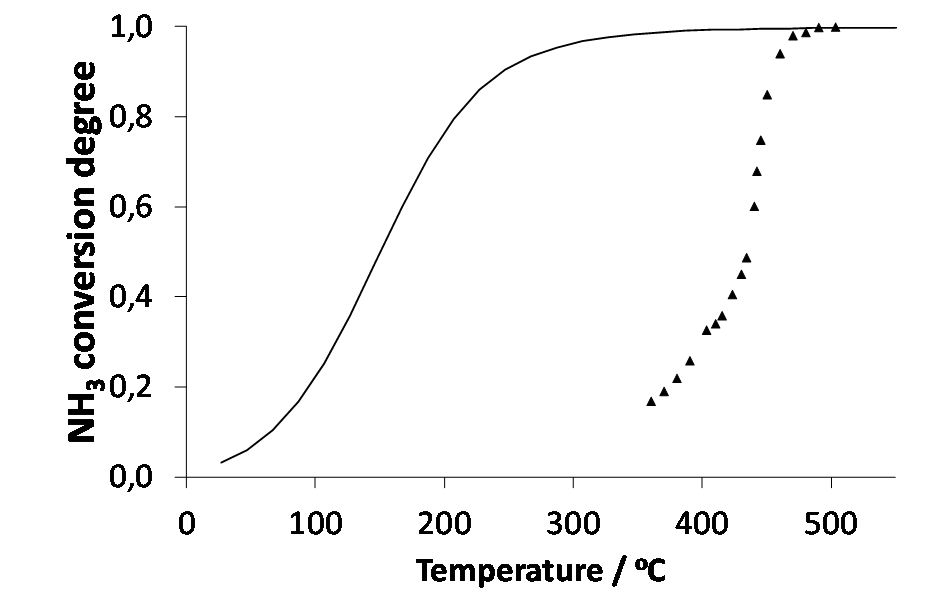

Supplement: S11 Fig — Ammonia conversion on the Pd/t-Ni catalyst compared to the thermodynamic equilibrium (solid line) at a flow rate of 2 dm3/h. (TIF) [file pone.0136805.s011.tif]

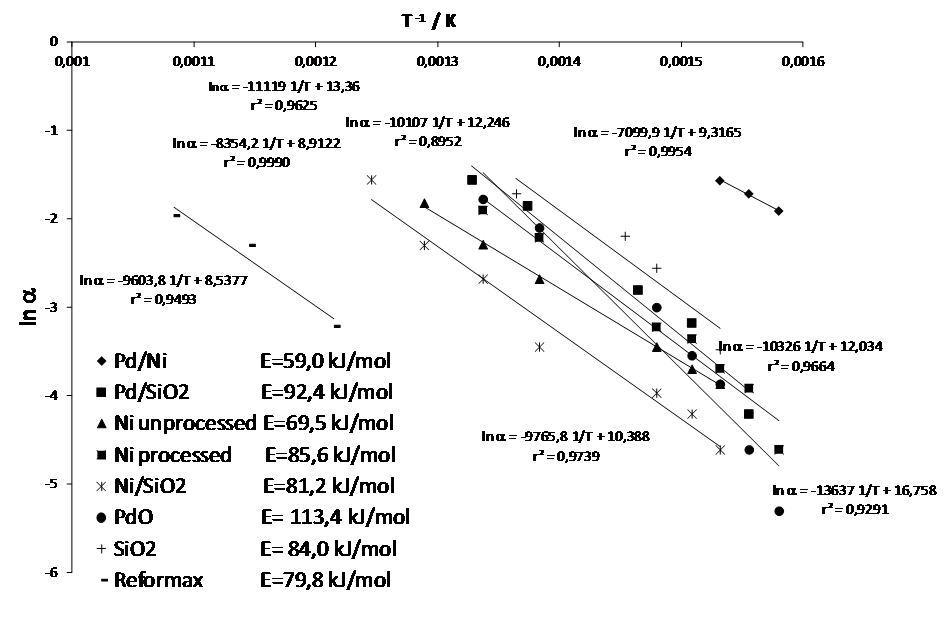

Supplement: S12 Fig — Activation energy calculated for the catalyst and reference systems that were tested. (TIF) [file pone.0136805.s012.tif]

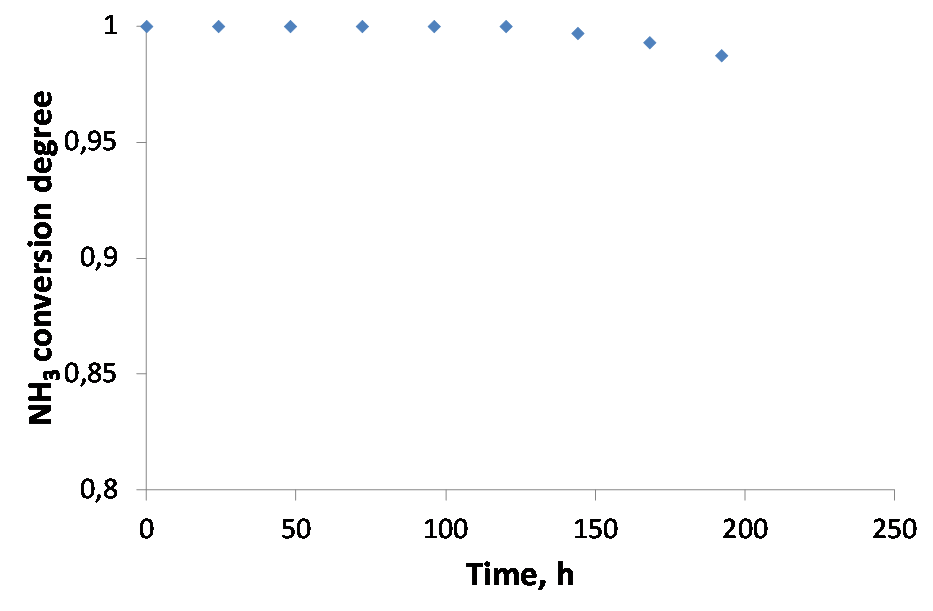

Supplement: S13 Fig — Conversion degree for a long duration experiment (Pd/t-Ni system). (TIF) [file pone.0136805.s013.tif]
