# Supplementary material for: Ni-Supported Pd Nanoparticles with Ca Promoter: A New Catalyst for Low-Temperature Ammonia Cracking
Source: PLoS One. 2015 Aug 26;10(8):e0136805. doi: 10.1371/journal.pone.0136805 (PMC4550460; doi:10.1371/journal.pone.0136805)
Supplement: S1 Text — Reagent list. (PDF) [file pone.0136805.s014.pdf]

Reagents

tetraethyl orthosilicate >= 99.0%; Sigma Aldrich
acetic acid >=99.8 puriss. p.a.; Sigma Aldrich
ammonium hydroxide solution puriss. p.a., 25% NH3 in H2O; Avantor Performance Materials Poland S.A.
ethyl alcohol absolut 99.8% pure p.a.; Avantor Performance Materials Poland S.A.
calcium chloride hexahydrate >=98.5% pure p.a.; Avantor Performance Materials Poland S.A.
sodium hydroxide >=98.8% pure p.a.; Avantor Performance Materials Poland S.A.
palladium (II) chloride anhydrous >=99.0% pure p.a.; Avantor Performance Materials Poland S.A.
nickel >=99.7% pure p.a.; or nickel technical grade B&K Bytom, Poland.
