# Supplementary material for: Ni-Supported Pd Nanoparticles with Ca Promoter: A New Catalyst for Low-Temperature Ammonia Cracking
Source: PLoS One. 2015 Aug 26;10(8):e0136805. doi: 10.1371/journal.pone.0136805 (PMC4550460; doi:10.1371/journal.pone.0136805)
Supplement: S2 Text — EDX Spectra. (PDF) [file pone.0136805.s015.pdf]

EDXRF spectra

The EDXRF spectra (Fig. S3) and quantitative analysis (the results are listed in Table S2) of the t-Ni system reveals the presence of major (Ni and Pd) and minor elements (Cr, Mn and Fe). In the preliminary study the Pd concentration was determined with Pd Lα line and Rh target X-ray tube operated at maximum voltage of 30 keV. The determined concentration of Pd (2.3 %) was much higher than the expected value (1.0%). It can be explained by the low energy of Lα line and the low information depth $d_{99\%}$ of ca 4 μm (see Table 1). In consequence, the Pd concentration is overestimated because Pd nanoparticles are located onto the surface of Ni particles. The reliable bulk composition can be obtained with Pd Kα line because of much higher energy and, in consequence, a much larger penetration range of X-rays compared to Pd Lα line ($d_{99\%}$ = 60-85 μm). This time, the determined Pd concentration is close to the expected value. It should be noted here that the Pd/Ni catalysts have very similar bulk composition before and after use.

Table S2. EDXRF analysis of nano-Pd/Ni catalyst (t-Ni) before (BR) and after reaction (AR). Results in % m/m.

Element	Pd Lα line; $d_{99\%}$ = 4 μm[a]		Pd Kα line; $d_{99\%}$ = 60-85 μm	
	BR	AR	BR	AR
Pd	2.43 ± 0.11	2.31 ± 0.14	1.23 ± 0.05	1.05 ± 0.04
Ca	2.13 ± 0.14	2.04 ± 0.18	2.21 ± 0.23	2.09 ± 0.26
Cr	0.27 ± 0.05	0.40 ± 0.04	0.37 ± 0.02	0.36 ± 0.02
Mn	0.055 ± 0.007	0.068 ± 0.006	0.074 ± 0.004	0.076 ± 0.005
Fe	2.41 ± 0.10	2.46 ± 0.10	2.52 ± 0.08	2.41 ± 0.09
Ni	92.7 ± 1.9	92.7 ± 2.2	93.6 ± 1.5	94.0 ± 2.0

[a] the information depth $d_{99\%}$ for element i that would yield 99% of the element intensity is given by the formula $d_{99\%}=4.6/\chi(E_0,E_i)\times\rho$, where ρ is the density of the sample and $\chi(E_0,E_i)=\mu(E_0)\csc(\phi_1)+\mu(E_i)\csc(\phi_2)$ is total mass-attenuation coefficient of the sample. $\mu(E_0)$ and $\mu(E_i)$ represent the mass attenuation coefficients of the sample at the primary E_0 and fluorescent radiation E_i (Pd Lα or Pd Kα line at 2.84 and 19.28 keV, respectively), ϕ_1 and ϕ_2 are the incidence and take-off angles, respectively.
