# Supplementary material for: Ni-Supported Pd Nanoparticles with Ca Promoter: A New Catalyst for Low-Temperature Ammonia Cracking
Source: PLoS One. 2015 Aug 26;10(8):e0136805. doi: 10.1371/journal.pone.0136805 (PMC4550460; doi:10.1371/journal.pone.0136805)
Supplement: S3 Text — Calculations of thermodynamic equilibrium. (PDF) [file pone.0136805.s016.pdf]

Calculations of thermodynamic equilibrium

For the reaction in gas phase:

$2\ NH_3 \leftrightarrow N_2 + 3H_2$

equilibrium constant at P = 0,1 MPa = idem is given by equation [1]

$$K = \frac{27}{16}\frac{\alpha_{eq}^4}{\left(1-\alpha_{eq}^2\right)^2} \tag{1}$$

which gives:

$$\alpha_{eq} = \frac{\left(\frac{16}{27}K\right)^{1/4}}{\left[1+\left(\frac{16}{27}K\right)^{1/2}\right]^{1/2}}$$

From the tables given by [2] we obtain:

lnK = 26.245 – (11824.7/T) , for 298.15 K$\leq$ T $\leq$ 900 K and $\Delta_r H$ = 98,3 kJ/mol (2)

References:

1. A. Mianowski, J. Szarawara, M. Minkina, Przem. Chem. 82 (2003) 1227
2. I. Barin, *Thermochemical Data of Pure Substances*, VCH, Wienheim, 1989
3. A. Mianowski, M. Tomaszewicz, T. Siudyga, T. Radko, Reac. Kinet. Mech. Cat. 111 (2014) 45
